# Morphology and molecular phylogeny of a new soil stichotrichid ciliate, *Circinella granulifera* sp. nov. (Alveolata, Ciliophora)

**DOI:** 10.3897/zookeys.1292.205482

**Published:** 2026-09-15

**Authors:** Lijian Liao, Hongyue Song, Xiaozhong Hu

**Affiliations:** 1 College of Fisheries, Ocean University of China (OUC), Qingdao 266003, China College of Fisheries, Ocean University of China (OUC) Qingdao China; 2 Key Laboratory of Evolution and Marine Biodiversity (Ministry of Education), Ocean University of China (OUC), Qingdao 266003, China Key Laboratory of Evolution and Marine Biodiversity (Ministry of Education), Ocean University of China (OUC) Qingdao China

**Keywords:** Ciliate, Hypotrichia, novel species, SSU rRNA gene, taxonomy

## Abstract

The morphology and molecular characterisation of a novel soil stichotrichid ciliate, *Circinella granulifera***sp. nov**., discovered in Ganzhou, southeastern China, were investigated using light microscopy of live and protargol-impregnated specimens, as well as ribosomal gene sequencing methods. The new species can be recognised by having a cell size of 115–130 × 10–15 µm *in vivo*, a vermiform body with a narrowly rounded anterior end and a tapered posterior portion that is consistently curved leftwards; a body only slightly dorsoventrally flattened in the frontal region; a single contractile vacuole positioned at about mid-body near the left margin; colourless cortical granules about 0.8 μm in diameter and arranged in rows of varying lengths; an adoral zone occupying about 1/10 of the body length and composed of 10 membranelles on average; three frontal membranelles in the distal portion which are separated by a distinct gap from ventral membranelles in the proximal portion; invariably three frontal cirri and one buccal cirrus; one frontoventral row of about seven cirri; one left and one right marginal row with 36–55 cirri and 48–70 cirri, respectively; one dorsal kinety with about 17 dikinetids; and on average 17 macronuclear nodules and two micronuclei. Phylogenetic analyses based on small subunit ribosomal RNA gene sequences showed that *Circinella granulifera***sp. nov**. clusters with *Kahliella matisi* with moderate to high support, suggesting that *Circinella* should be assigned to or closely related to the family Kahliellidae Tuffrau, 1979.

## Introduction

Hypotrichs (subclass Hypotrichia Stein, 1859) belong to the most complex and highly differentiated ciliate group, and there are over 1,200 species distributed worldwide in a variety of habitats, such as soil, freshwater, brackish water, and seawater (Kahl 1932; Berger 2008, 2011; Lynn 2008; Kumar and Foissner 2016; Hu et al. 2019; Paiva 2020; Foissner and Berger 2021). In recent decades, many new taxa have been reported, indicating that the diversity of this ciliate lineage is far greater than previously expected (e.g. Foissner et al. 2002; Kumar and Foissner 2016; Liao et al. 2025; Omar et al. 2025; Wang et al. 2025). Although hypotrichs have attracted increasing attention in recent years, they remain one of the most taxonomically confused group, especially the stichotrichids (order Stichotrichida Fauré-Fremiet, 1961) within the subclass. Due to insufficient faunistic studies, a large number of species remain undiscovered, and the diversity of soil stichotrichids, in particular, is still severely underestimated (Berger 2001; Kumar and Foissner 2016).

The slender and rare genus *Circinella* was established by Foissner (1994) with *C. arenicola* as the type species. Various researchers hold differing views on the family classification of this genus. Foissner (1994) provisionally classified *Circinella* in the Cladotrichidae Small & Lynn, 1985 due to the apokinetal origin and development of the oral apparatus and ciliature of the opisthe and the lack of frontoterminal, midventral, transverse, and caudal cirri, a combination of features generally matching the very vague diagnosis of the cladotrichids. Eigner (1997) removed *Circinella* from the Cladotrichidae and placed it in the Orthoamphisiellidae Eigner, 1997. Shi (1999), Lynn and Small (2002), and Jankowski (2007) classified *Circinella* in the Amphisiellidae Jankowski, 1979, which was accepted by Adl et al. (2019). Lynn (2008) put it as incertae sedis in the amphisiellids. Berger (2011) recognised *Circinella* as incertae sedis in the family Gonostomatidae Small & Lynn, 1985. *Circinella* can be distinguished from other genera within the families mentioned above by its very slender, worm-like body shape, very short and bipartite adoral zone of membranelles, with one frontoventral row originating from single anlage, and only one marginal cirral row on each side of cell. To date, three species of *Circinella* have been identified: *C. arenicola*, *C. vettersi* (Berger & Foissner, 1989) Foissner 1994, and *C. filiformis* (Foissner, 1982) Foissner 1994.

In the present study, we describe a new soil ciliate discovered in the surface-soil layer in Ganzhou, southeastern China. Based on morphological and morphometric data, we classified it as a new species of *Circinella*. Moreover, its phylogenetic position was determined using the newly obtained small subunit ribosomal RNA gene sequence. We also briefly discuss the family classification of the genus *Circinella*.

## Material and methods

### Sampling and cultivation

*Circinella granulifera* sp. nov. was isolated from a soil sample collected from the surface (up to 10 cm depth, including leaf litter and moss) irrigation ditches for farmland (26°23'30.55"N, 115°44'27.06"E) in Ganzhou, Jiangxi Province, China on 12 February 2025. At the time of collection, the air temperature was approximately 15 °C. The soil percolate had a salinity of <1‰ (measured with an ATAGO MASTER–S/Milla, cat. no. 2491) and a pH of approximately 7, as determined by pH indicator papers. After being left to air-dry in the laboratory for two weeks, the soil sample was rehydrated with natural mineral water (Volvic, Danone) to trigger the excystment of ciliates. This was conducted using the non-flooded Petri dish method of Foissner et al. (2002). Non-clonal cultures were maintained for two weeks at room temperature (approximately 25 °C) in Petri dishes using natural mineral water (Volvic, Danone) with pre-sterilised rice grains to support bacterial growth as nutritional source for the ciliates.

### Morphology

Live cells were isolated from the Petri dishes using micropipettes and observed under bright field and differential interference contrast illumination with a digital camera mounted on the light microscopes (Zeiss AXIO Imager D2, Jena, Germany and Olympus BX53, Tokyo, Japan) at a magnification of 100–1,000×. The nuclear apparatus and infraciliature were revealed using the protargol staining method according to Wilbert (1975). Protargol powder was synthesised following the protocol of Pan et al. (2013). Measurements and counts on protargol-impregnated specimens were performed at a magnification of 1,000×. Drawing of a live specimen was made based on photographic records, while stained cell morphologies were captured using a camera setup. Classification and terminology mainly follow Berger (2011).

### DNA extraction, PCR amplification, and sequencing

Target ciliate was picked out individually from the raw culture (without other ciliates recognised during the study period) using micropipettes under a stereomicroscope. The cells were washed at least five times with syringe-filtered natural mineral water (Millex-GP Filter, 0.22 μm PES membrane) to eliminate potential contaminations. Subsequently, single washed cell was transferred to a 1.5 mL micro-centrifuge tube with a minimal volume of water. Genomic DNA extraction was carried out using the DNeasy Blood and Tissue Kit (Qiagen, Germany) according to the manufacturer’s instructions. PrimeSTAR Max Premix (2×) DNA polymerase (Takara) was employed to minimise experimental errors associated with the polymerase chain reaction (PCR). Extracted DNA was amplified using the PCR primers 18S-F (5'- AAC CTG GTT GAT CCT GCC AGT-3') and 18S-R (5'-TGA TCC TTC TGC AGG TTC ACC TAC-3') (Medlin et al. 1988). The optimised PCR conditions were as follows: initial denaturation step at 98 °C for 30 s, followed by 35 cycles of denaturation at 98 °C for 10 s, annealing at 55 °C for 15 s, and extension at 72 °C for 10 s. After 35 cycles, the final extension step was run at 72 °C for 5 min. The PCR products was sequenced bidirectionally by the Sangon Biological Technology Company (Qingdao, China) using primers 18S-F, 18S-R, and three internal primers 900F (5'- CGA TCA GAT ACC GTC CTA GT -3'), 900R (5'- ACT AGG ACG GTA TCT GAT CG-3'), and Pro B (5'- GGT TAA AAA GCT CGT AGT-3') (Zhao et al. 2025). The sequence was assembled using Seqman v. 7.1.0 (DNA Star).

### Phylogenetic analyses

To determine the phylogenetic position of the new species, the newly obtained SSU rRNA gene sequence was aligned with 73 other hypotrich and four euplotid sequences retrieved from the National Center for Biotechnology Information (NCBI) database. Four euplotids, namely *Apodiophrys ovalis* (GU477634), *Diophrys scutum* (JF694040), *Paradiophrys zhangi* (FJ870076), and *Uronychia multicirrus* (EU267929), were selected as the outgroup species. All sequences were aligned using the MAFFT package on the web server of the European Bioinformatics Institute (https://www.ebi.ac.uk/jdispatcher/msa/mafft). The alignment results were manually edited using the program BioEdit v. 7.2.5 (Hall 1999). Primer sequences were manually eliminated, and both ends of the alignment were then trimmed. The final alignment used for phylogenetic analyses comprised 78 taxa and 1,796 nucleotide sites. Maximum likelihood (ML) analysis was conducted using IQ-TREE v. 3.0 with 10,000 ultrafast bootstrap replicates. The TIM2 + F + I + R4 model was selected as the best-fit model based on the Bayesian Information Criterion (BIC) (Wong et al. 2026). Bayesian inference (BI) analysis was performed using MrBayes v. 3.2.7a on the CIPRES Science Gateway (ACCESS v. 3.2.7) (Ronquist et al. 2012) with the GTR + I (0.5752) + G (0.4635) model chosen by the Akaike Information Criterion in MrModeltest v. 2.2 (Nylander 2004). Four Markov chain Monte Carlo (MCMC) simulations were run for one million generations with a sampling frequency of 100 and a burn-in of 25,000 trees. The remaining trees were used to generate a consensus tree and calculate the posterior probabilities with a majority rule consensus. MEGA v. 11.0.13 (Kumar et al. 2018) and Seaview v. 5.0.5 (Gouy et al. 2010) were used to visualise the topologies of phylogenetic trees.

Thresholds of phylogenetic support were categorised as follows: ML bootstrap values ≥ 95%, 71–94%, 50–70%, and < 50% were regarded as high, moderate, low, and no support, respectively (Hillis and Bull 1993). Bayesian nodal support was assessed using posterior probabilities, with values ≥0.95 indicating high support and those <0.95 indicating low support (Alfaro et al. 2003). Accession numbers are provided after the species names in the phylogenetic tree.

## Results

### Taxonomy

**Class Spirotrichea Bütschli, 1889**

**Subclass Hypotrichia Stein, 1859**

**Order Stichotrichida Fauré-Fremiet, 1961**

**Genus *Circinella* Foissner, 1994**

#### Circinella granulifera sp. nov.

Taxon classificationAnimaliaMucoralesSyncephalastraceae

94818007-0B7D-5D95-978B-956B40D6BDD3

https://zoobank.org/3770DD4D-12C2-4E3B-8234-E263431C5C4D

**Figure 1.**
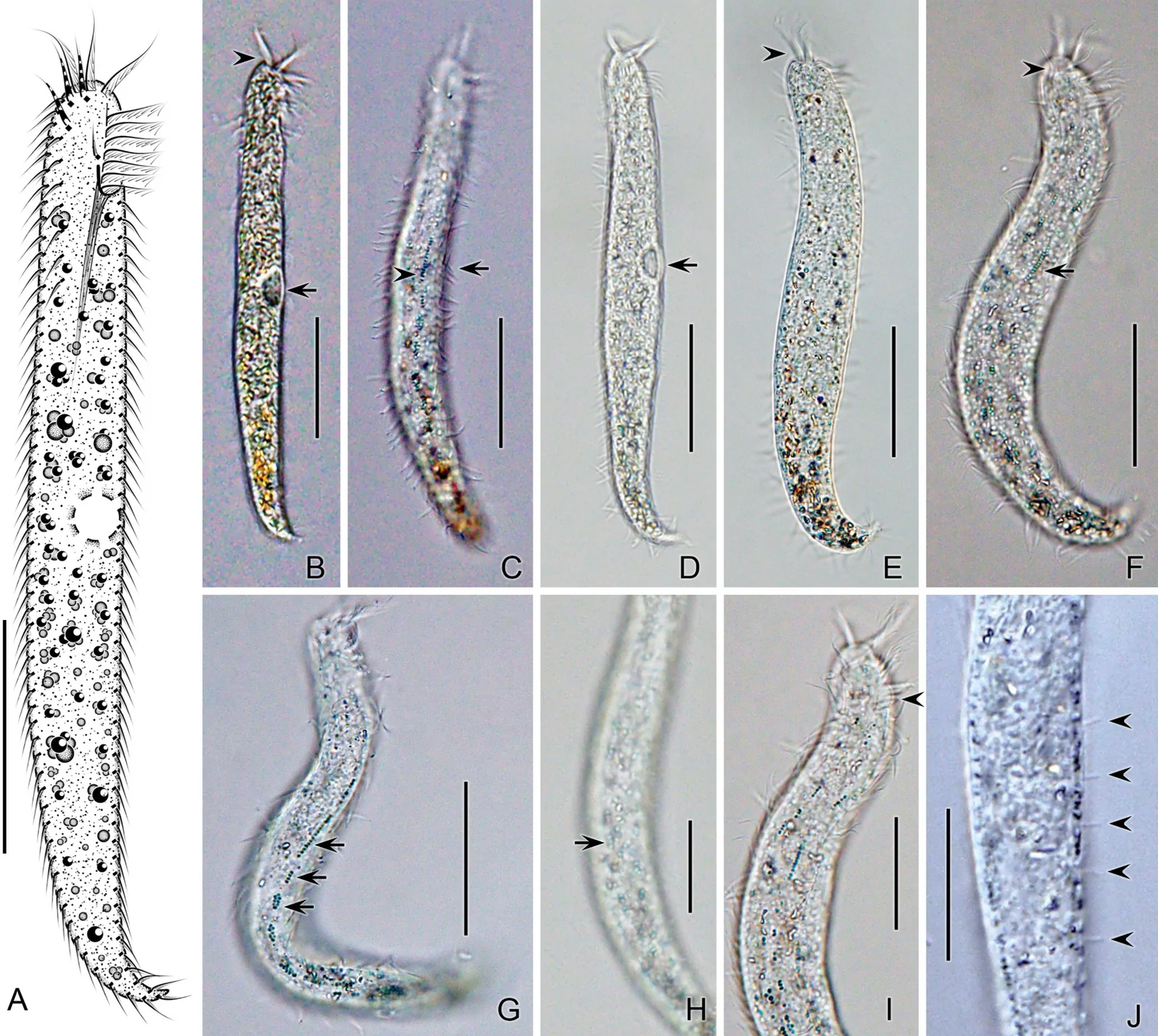
*Circinella granulifera* sp. nov. from life. **A**. Ventral view of a representative individual; **B, D**. Ventral view showing the body shape and contractile vacuole (arrows), arrowhead indicates the frontal membranelles; **C**. Ventral view, arrow denotes the marginal cirri and arrowhead points to the cortical granules; **E**. Ventral view showing some highly refractive granules in posterior portion, arrowhead denotes the frontal membranelles; **F, G**. Ventral views of slightly compressed individual, arrowhead indicates the frontal cirrus and arrows point to the cortical granules; **H**. Dorsal view, arrow indicates the cortical granules; **I**. Detail of the anterior body portion, arrowhead denotes the ventral membranelles; **J**. Detail of the dorsal bristles (arrowheads). Scale bars: 30 μm (**A–G**); 15 μm (**H–J**).

**Figure 2.**
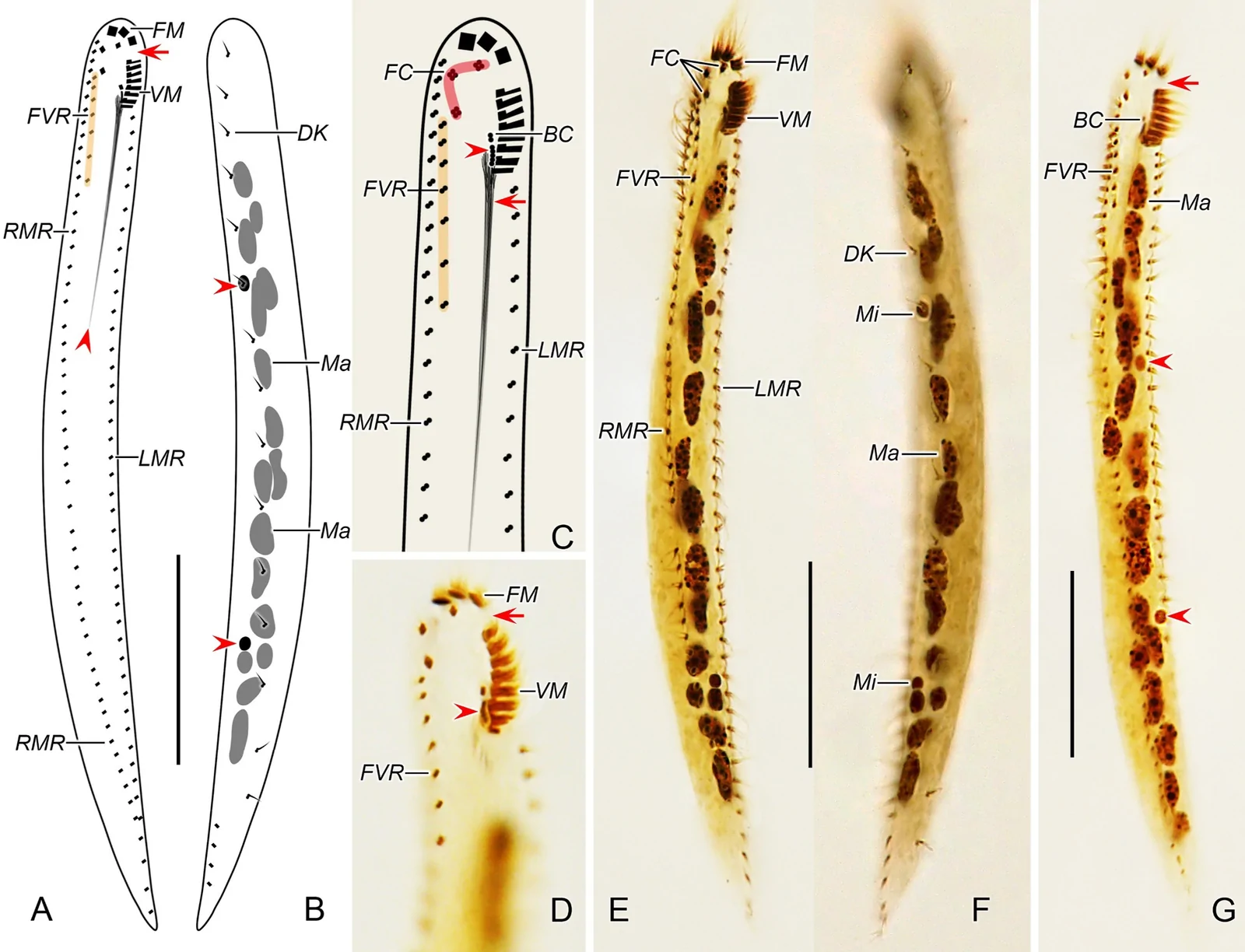
*Circinella granulifera* sp. nov. after protargol impregnation. **A**. Holotype, ventral view showing infraciliature and nuclear apparatus, arrow marks gap in adoral zone of membranelles, arrowhead points to pharyngeal fibres; **B**. Holotype, dorsal view, arrowheads indicate the micronuclei; **C**. Detailed ventral view of the cell anterior region, arrow denotes the pharyngeal fibres and arrowhead indicates the undulating membrane; **D**. Ventral view of the anterior portion of cell, arrowhead indicates the undulating membrane and arrow points to the gap in adoral zone of membranelles; **E**. Holotype, ventral view; **F**. Holotype, dorsal view; **G**. Paratype, ventral view, arrow indicates gap in adoral zone of membranelles and arrowheads denote micronuclei. Abbreviations: BC, buccal cirrus; DK, dorsal kinety; FC, frontal cirri; FM, frontal adoral membranelles; FVR, frontoventral row; LMR, left marginal row; Ma, macronuclear nodules; Mi, micronuclei; RMR, right marginal row; VM, ventral adoral membranelles. Scale bars: 30 μm (**A, B, E–G**).

Figs 1A–J, 2A–G; Table 1

##### Diagnosis.

Body 115–130 × 10–15 μm *in vivo*, vermiform. On average, 17 macronuclear nodules globular to elongated ellipsoidal, distinctly separated and arranged in a row, and 2–5 micronuclei. Single contractile vacuole located at mid-body near left margin. Cortical granules colourless, about 0.8 μm across, arranged in rows of varying lengths. Adoral zone about 10% of body length, composed of 10 membranelles on average; three frontal membranelles separated by a distinct gap from about seven ventral membranelles. Three conspicuous frontal cirri and one buccal cirrus. One short and indistinct undulating membrane composed of five monokinetids, located right of proximal portion of adoral zone. Frontoventral row extends beyond level of adoral zone, composed of an average of seven cirri. One left and one right marginal row with 36–55 and 48–70 marginal cirri, respectively. One almost bipolar dorsal kinety with about 17 dikinetids.

**Table 1.** Morphometric characterisation of *Circinella granulifera* sp. nov.

Characteristi^c^*	**HT**	**Min**	**Max**	**Mean**	**Med**	**SD**	**CV**	***n***
Body length (μm)	130.4	107.1	183.7	139.1	134.7	22.6	16.2	21
Body width (μm)	11.5	9.0	17.5	11.8	11.4	2.1	17.6	21
Body length:width ratio	11.30	9.81	15.25	11.93	11.87	1.37	11.5	21
Macronuclear nodules, number	16	14	23	16.9	16	2.6	15.4	21
Anterior macronuclear nodule length	9.1	6.1	12.2	8.3	8.2	1.8	21.8	21
Anterior macronuclear nodule width	3.5	2.3	4.5	3.1	3.1	0.5	15.4	21
Micronuclei, number	2	2	5	2.4	2	0.7	30.7	21
Anterior micronucleus length (μm)	2.5	2.1	3.4	2.6	2.6	0.4	15.8	21
Anterior micronucleus width (μm)	1.8	1.3	2.0	1.7	1.7	0.1	8.7	21
Adoral zone of membranelles length (μm)	13.1	11.2	14.6	12.9	13.1	0.8	6.5	21
Adoral zone length, % of body length	10.1	7.3	12.0	9.5	9.3	1.4	15.2	21
Adoral membranelles, number in distal portion	3	3	3	3.0	3	0	0	21
Adoral membranelles, number in proximal portion	7	7	8	7.2	7	0.4	6.0	21
Adoral membranelles, total number	10	10	11	10.2	10	0.4	4.3	21
Frontal cirri, number	3	3	3	3.0	3	0	0	21
Buccal cirrus, number	1	1	1	1.0	1	0	0	21
Frontoventral row, number of cirri	7	5	10	7.2	7	1.0	14.3	21
Right marginal row, number of cirri	51	48	70	57.9	58	7.5	13.0	21
Left marginal row, number of cirri	44	36	55	45.2	45	5.3	11.6	21
Dorsal kineties, number	1	1	1	1.0	1	0	0	21
Dikinetids in dorsal kinety, number	16	14	20	16.8	17	1.3	7.7	21
Largest adoral membranelle, length of base (μm)	3.4	2.2	3.8	2.9	2.9	0.4	14.3	21
Anterior body end to buccal cirrus, distance	9.1	6.4	10.5	9.0	9.3	1.0	11.6	21
Anterior body end to anterior end of undulating membrane, distance (μm)	10.0	8.2	11.9	10.0	10.1	1.0	9.5	21
Anterior body end to anterior end of frontoventral row, distance (μm)	7.7	5.0	9.3	7.6	7.9	1.3	16.4	21
Anterior body end to first macronuclear nodule, distance (μm)	16.8	9.5	22.0	15.7	15.2	3.5	22.4	21

*All data are based on protargol-impregnated specimens and measurements in μm. Abbreviations: CV, coefficient of variation in %; HT, holotype specimen; Max, maximum; Mean, arithmetic mean; Med, median; Min, minimum; *n*, number of specimens examined; SD, standard deviation.

##### Type locality.

Soil sample from irrigation ditches in farmland in Ganzhou, southeastern China (26°23'30.55"N, 115°44'27.06"E) (for details, see Material and methods).

##### Type materials.

One slide with protargol-impregnated specimens (registration no. LLJ2025021203/1) containing the holotype (circled with black ink on the back of the slide; Fig. 2A, B, E, F) and three paratype slides (registration nos LLJ2025021203/2–4) are deposited in the Laboratory of Systematic Taxonomy, College of Fisheries, OUC, Qingdao, China.

##### Etymology.

The species-group name *granulifera* (Latin adjective; bearing granules) alludes that the present species has conspicuous cortical granules.

##### Description.

Body size 115–130 × 10–15 μm (*n* = 7) *in vivo*, 107–184 × 9–17 μm (average 139 × 12 μm) in protargol preparations; length:width ratio on average 12:1 (Table 1). Body vermiform, anterior end narrowly rounded, posterior portion tapered and consistently curved leftwards; body slightly dorsoventrally flattened solely in frontal region (Fig. 1A–H). Pellicle flexible. Cortical granules colourless, about 0.8 μm across, arranged in rows of varying lengths (Fig. 1C, F–I). Cytoplasm colourless, with lipid globules, many irregular crystals, and some highly refractive granules in posterior portion (Fig. 1B, C, E, F). Macronuclear nodules 14–23 (on average 17) in central body portion slightly left of midline; nodules never connected, globular to elongated ellipsoidal; anterior nodule about 8 × 3 μm in protargol preparations. Micronuclei 2–5, usually two bluntly ellipsoidal or ellipsoidal, with one in anterior and the other in posterior body portion; anterior micronucleus about 3 × 2 μm for stained specimens (Fig. 2B, E–G). Single contractile vacuole at mid-body near left margin of the cell, approximately 6 μm in diameter when fully extended, pulsating at intervals of about 11 s (Fig. 1A, B, D). Craws slowly on the bottom of a petri dish or other substrates.

Bipartite adoral zone occupying about 10% of body length, composed of 10 or 11 membranelles; invariably three slightly enlarged frontal membranelles in distal portion, separated from ventral membranelles in proximal portion by a distinct gap (Figs 1A, 2A, 2C–E, 2G). Bases of largest membranelles only about 5 μm long in life and about 3 μm long in protargol preparations (Figs 1A, 1F, 1I, 2A C–E, 1G). One short, indistinct undulating membrane composed of five monokinetids, located right of proximal portion of adoral zone (Fig. 2A, C–E, G). Buccal field very small and flat, and thus inconspicuous. Pharyngeal fibres conspicuously strong, about 35 μm long, almost anterior 1/3 of cell (Figs 1A, 2A, 2C, 2E).

Cirral pattern rather stable. Three slightly enlarged frontal cirri, each comprised of four kinetosomes, which are located near the frontal membranelles except for the third one displaced, positioned below the right frontal cirrus (Figs 1F, 2A, 2C–E, 2G). Single buccal cirrus, composed of only two kinetosomes, mostly positioned ahead of undulating membrane (Figs 1A, 2 A, 1C–E, 1G), very rarely (one out of 21 specimens examined) right of anterior end of undulating membrane. Frontoventral cirral row composed of 5–10 (on average seven) cirri, right of and behind the posteriormost frontal cirrus, and beyond proximal portion of adoral zone (Figs 1A, 2A, 2C–E, 2G). Frontoterminal, postoral and pretransverse ventral, and transverse cirri lacking. One right and one left marginal row, consisting of 48–70 and 36–55 cirri, respectively. Right marginal row commences near the frontal membranelle at the distal end of adoral zone, while left marginal row left of proximal portion of adoral zone. Both terminates at rear cell end. with cilia about 6 μm long (Figs 1A, 1C, 2A, 2C–E, 2G). All frontoventral and marginal cirri very fine, composed of two kinetosomes each (Fig. 2C).

One bipolar dorsal kinety, with 14–20 (17 on average) equidistant dikinetids; dorsal bristles about 3 μm long *in vivo* (Figs 1J, 2B, 2F).

### Phylogenetic analyses

The SSU rRNA gene sequence of *Circinella granulifera* sp. nov. has been deposited in GenBank under the accession number PZ538963. The length and G + C content of the new sequence are 1,729 bp and 45.69%, respectively.

**Figure 3.**
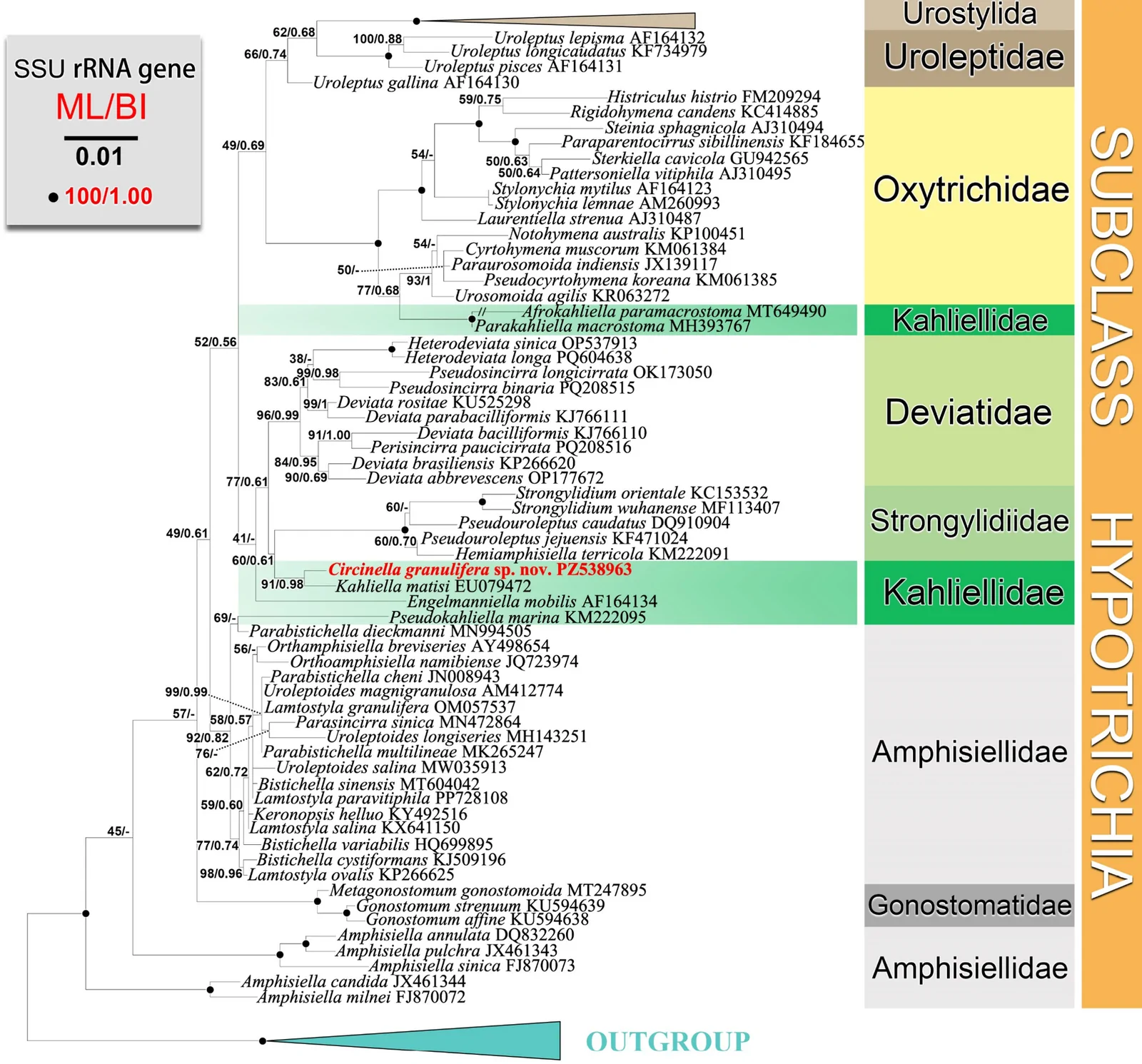
Maximum-likelihood (ML) tree based on SSU rRNA gene sequences showing the position of *Circinella granulifera* sp. nov. (in red). Numbers at nodes represent the ML bootstrap support and BI posterior probability values. Fully supported (100/1.00) branches are marked with filled circles. Asterisks indicate disagreement between the BI tree and the ML tree. All branches are drawn to scale. The scale bar corresponds to one substitution per 100 nucleotide positions.

Phylogenetic trees constructed from ML and BI analyses exhibit analogous topologies, albeit with some variations in support values between the methods. Consequently, solely the ML tree topology is presented here, with support values from both algorithms displayed. In the tree, *Circinella granulifera* sp. nov. clusters with *Kahliella matisi* Vďačný et al., 2010, forming a reliably supported clade (91% ML / 0.98 BI), which nests within a clade that also contains five species from the family Strongylidiidae Fauré-Fremiet, 1961 and 10 species from the family Deviatidae Foissner, 2016 (Fig. 3).

Accordingly, seven species from the families Deviatidae and Strongylidiidae mentioned above, as well as *Kahliella matisi* were selected as molecularly related taxa of *Circinella granulifera* sp. nov. A comparison of SSU rRNA gene sequences among them was performed. *Circinella granulifera* sp. nov. differs from these eight species by 10–56 nucleotide positions corresponding to 96.3–99.3% sequence similarity (Table 2).

**Table 2.** Sequence similarities (below diagonal) and nucleotide differences (above diagonal) among *Circinella granulifera* sp. nov. and eight molecularly related species.

**Sequence**s	1	2	3	4	5	6	7	8	9
1. *Circinella granulifera* sp. nov. PZ538963	—	10	29	37	36	23	52	56	35
2. *Kahliella matisi*EU079472	0.993	—	30	38	37	25	53	53	40
3. *Deviata abbrevescens*OP177672	0.981	0.980	—	31	31	17	55	58	45
4. *Heterodeviata sinica*OP537913	0.976	0.975	0.979	—	35	31	57	62	52
5. *Pseudosincirra longicirrata*OK173050	0.976	0.976	0.979	0.977	—	32	56	63	51
6. *Perisincirra paucicirrata*PQ208516	0.985	0.983	0.988	0.979	0.979	—	55	55	42
7. *Strongylidium orientale*KC153532	0.966	0.965	0.964	0.963	0.963	0.964	—	38	25
8. *Pseudouroleptus caudatus*DQ910904	0.963	0.965	0.962	0.959	0.959	0.964	0.975	—	28
9. *Hemiamphisiella terricola*KM222091	0.977	0.974	0.970	0.966	0.966	0.972	0.983	0.981	—

## Discussion

### Comparison of *Circinella granulifera* sp. nov. with its congeners

Prior to this study, the genus *Circinella* contains only three species.

*Circinella arenicola* differs from *C. granulifera* sp. nov. in body length *in vivo* (more than 250 μm vs 115–130 μm), lack of cortical granules (vs present), number of adoral membranelles (15–27 vs 10 or 11), number of cirri in frontoventral row (18–37 vs 5–10), number of macronuclear nodules (46–102 vs 14–23), number of contractile vacuoles (about 10 vs 1), and number of dorsal kineties (3 or 4 vs 1) (Foissner 1994).

*Circinella vettersi* differs from *C. granulifera* sp. nov. in number of adoral membranelles (7–9 vs 10 or 11), lack of cortical granules (vs present), number of contractile vacuoles (about 6 vs 1), and number of dorsal kineties (3 vs 1) (Berger and Foissner 1989; Foissner 1994).

*Circinella filiformis* closely resembles *C. granulifera* sp. nov. in body shape and infraciliature but can be distinguished by the average body length: width ratio (15 vs 12), length of adoral zone of membranelles (6–9 μm vs ca 11–15 μm), the lack of cortical granules (vs present), and number and position of contractile vacuoles (2, at about at 25% and 65% of body length vs 1, at mid-body) (Foissner 1994).

### Comparison of *Circinella granulifera* sp. nov. with three slender species of *Hemisincirra*

Although species of *Circinella* and *Hemisincirra* can be clearly separated by their relative lengths of the frontoventral cirral rows, several most similar *Hemisincirra* species are almost indistinguishable from *Circinella* in life. In terms of slender and worm-shaped body with more than eight macronuclear nodules, *Circinella granulifera* sp. nov. should be compared with three *Hemisincirra* species, namely, *Hemisincirra interrupta* Foissner, 1982, *H. vermicularis* Hemberger, 1985, and *H. rariseta* Foissner, Agatha & Berger, 2002.

*Hemisincirra interrupta* differs from *C. granulifera* sp. nov. by the lack of cortical granules (vs present), number of adoral membranelles (12–16 vs 10 or 11), and number of macronuclear nodules (on average 30 vs 17) (Foissner 1982; Berger 2008).

*Hemisincirra vermicularis* differs from *C. granulifera* sp. nov. by the number of macronuclear nodules (on average 10 vs 17), and number of contractile vacuoles (4 vs 1) (Hemberger 1985).

*Hemisincirra rariseta* differs from *C. granulifera* sp. nov. by the number of adoral membranelles (15–17 vs 10 or 11), lack of cortical granules (vs present), number of left marginal cirri (21–29 vs 36–55), number of right marginal cirri (21–36 vs 48–70), and number of dorsal kineties (2 vs 1) (Foissner et al. 2002).

### Systematic placement

In our phylogenetic analyses, *Circinella granulifera* sp. nov. grouped with *Kahliella matisi*, the only species with available molecular sequence within the type genus *Kahliella* (family Kahliellidae Tuffrau, 1979), with moderate to high support (91% ML / 0.98 BI). Tuffrau (1979) established the family Kahliellidae primarily based on the following diagnostic features: (i) absence of transverse cirri, (ii) presence of longitudinal rows of frontoventral cirri, (iii) more or less distinct marginal rows, and (iv) well-developed frontal cirri. Since the genus *Circinella* appears to fit this diagnosis, making its placement within the Kahliellidae seems reasonable. However, Berger (2011) noted that preservation of parental cirri and/or dorsal bristles appears to be a feature generally used to characterise the kahliellids. However, in the type species of *Circinella*, *C. arenicola*, no such structures are retained during interphase. Thus, assigning *Circinella* to Kahliellidae appears questionable.

In this study, we first provided the molecular data for the genus *Circinella* and revealed the sister-group relationship between *Circinella* and *Kahliella*. Given the morphological similarities (absence of transverse cirri, presence of more or less enlarged frontal cirri, presence of longitudinal row of frontoventral cirri, rows with widely spaced cirri and lack of dorsal kinety fragmentation) between the two genera and the close phylogenetic relationship of *C. granulifera* sp. nov. with *Kahliella matisi*, we provisionally assign *Circinella* to the family Kahliellidae. It should be noted, however, that SSU rRNA gene sequences of the type species for both genera are currently unavailable, leaving their precise phylogenetic positions unresolved. Consequently, we do not rule out the possibility that *Circinella* may represent a new family and serve as the sister group to Kahliellidae. Future studies integrating morphological, morphogenetic, and molecular phylogenetic evidence are required to test this hypothesis.

## Supplementary Material

XML Treatment for Circinella granulifera

## Additional information

### Artificial Intelligence (AI) use

The authors accept full responsibility for the content of the manuscript, including the disclosure of any use of AI.

No AI tools were used in the preparation of this manuscript.

### Author ORCIDs

L. Liao https://orcid.org/0009-0004-8290-8692

H. Song https://orcid.org/0009-0002-5427-5766

X. Hu https://orcid.org/0000-0003-2647-9980

### Data availability

All of the data that support the findings of this study are available in the main text.
